# Prebiotics Supplementation Impact on the Reinforcing and Motivational Aspect of Feeding

**DOI:** 10.3389/fendo.2018.00273

**Published:** 2018-05-29

**Authors:** Anne-Sophie Delbès, Julien Castel, Raphaël G. P. Denis, Chloé Morel, Mar Quiñones, Amandine Everard, Patrice D. Cani, Florence Massiera, Serge H. Luquet

**Affiliations:** ^1^Université Paris Diderot, Sorbonne Paris Cité, Unité de Biologie Fonctionnelle et Adaptative, CNRS UMR 8251, Paris, France; ^2^Walloon Excellence in Life Sciences and Biotechnology (WELBIO), Metabolism and Nutrition Research Group, Louvain Drug Research Institute, Université catholique de Louvain, Brussels, Belgium; ^3^Laboratoire de Recherche Nutritionnelle KOT CEPRODI SA, Paris, France

**Keywords:** food intake, hedonic and motivational component, dopaminergic system, prebiotic, reward

## Abstract

Energy homeostasis is tightly regulated by the central nervous system which responds to nervous and circulating inputs to adapt food intake and energy expenditure. However, the rewarding and motivational aspect of food is tightly dependent of dopamine (DA) release in mesocorticolimbic (MCL) system and could be operant in uncontrolled caloric intake and obesity. Accumulating evidence indicate that manipulating the microbiota–gut–brain axis through prebiotic supplementation can have beneficial impact of the host appetite and body weight. However, the consequences of manipulating the implication of the microbiota–gut–brain axis in the control motivational and hedonic/reinforcing aspects of food are still underexplored. In this study, we investigate whether and how dietary prebiotic fructo-oligosaccharides (FOS) could oppose, or revert, the change in hedonic and homeostatic control of feeding occurring after a 2-months exposure to high-fat high-sugar (HFHS) diet. The reinforcing and motivational components of food reward were assessed using a two-food choice paradigm and a food operant behavioral test in mice exposed to FOS either during or after HFHS exposure. We also performed mRNA expression analysis for key genes involved in limbic and hypothalamic control of feeding. We show in a preventive-like approach, FOS addition of HFHS diet had beneficial impact of hypothalamic neuropeptides, and decreased the operant performance for food but only after an overnight fast while it did not prevent the imbalance in mesolimbic markers for DA signaling induced by palatable diet exposure nor the spontaneous tropism for palatable food when given the choice. However, when FOS was added to control diet after chronic HFHS exposure, although it did not significantly alter body weight loss, it greatly decreased palatable food tropism and consumption and was associated with normalization of MCL markers for DA signaling. We conclude that the nature of the diet (regular chow or HFHS) as well as the timing at which prebiotic supplementation is introduced (preventive or curative) greatly influence the efficacy of the gut–microbiota–brain axis. This crosstalk selectively alters the hedonic or motivational drive to eat and triggers molecular changes in neural substrates involved in the homeostatic and non-homeostatic control of body weight.

## Introduction

Obesity and corollary pathologies, such as dyslipidemia, diabetes, and cardiovascular diseases are spreading in both developed and developing countries as a result of increased accessibility to energy-dense food associated with a general decrease in physical activity and energy expenditure (1). Whereas some genetic loci were clearly identified and extensively studied as monogenic causes for obesity, it is widely accepted that the metabolic syndrome is in essence a multifactorial disease that encloses a complex network of molecular, cellular, and physiologic alterations (1, 2). Understanding the complex pathology of the metabolic syndrome will be critical in shaping effective preventive and therapeutic strategies. However, despite the encouraging results obtained through pharmacological and surgical interventions, no effective anti-obesity treatment with long-lasting effects on body weight is nowadays available.

Proper energy balance is insured by the ability of the central nervous system to integrate nervous and circulating signals that reflect nutritional status to produce adaptive metabolic and behavioral responses aiming at maintaining body weight within a physiological narrow range (3). In addition, the rewarding aspects of energy-dense food largely depends on dopamine (DA) release from dopaminergic neurons in the ventral tegmental area that project to limbic regions, notably the prefrontal cortex and the nucleus accumbens (NAcc) (4, 5). This neural substrate referred as to mesolimbic “reward” circuit is instrumental in the encoding of both the volume of desire, i.e., “motivation” and the hedonic aspect, i.e., “liking” in food rewards (6).

Hence, the complex behavioral sequence leading to food intake results from the integration of metabolic needs but also reinforcing aspects of food. Multiple lines of evidence suggest that high-fat feeding and obesity *per se* can promote long-lasting adaptations in both hypothalamic and limbic regions thus leading to increased vulnerability to over-consume energy-dense food. In turn, such vulnerability can promote aberrant behaviors (7, 8) in which the reward becomes the primary driving force to consume energy-dense food (9–12). For instance, emerging theories suggest that chronic exposure to palatable food might impair the proper encoding of reward and, similar to drug of abuse, lead to desensitization of the DA mesolimbic system, and ultimately promote craving and addictive-like consummatory behavior (4, 6).

However, despite similar exposure to palatable and hypercaloric food, the development of eating-habits dissociated from actual homeostatic needs does not occur in every individual, suggesting differential degrees of vulnerability. In that regard, the microbiota–gut–brain axis has recently emerged as a key regulator of brain structures involved in stress-like responses (13, 14) together with resilience to high-fat-induced body weight gain (15). Dietary prebiotic such as the soluble fibers fructo-oligosaccharides (FOS) represents selectively fermented compounds that promote changes in the activity and composition of the gut microbiota, that are associated with a wide spectrum of beneficial effects including reduced appetite (16–20), decreased body weight (15), improved glucose metabolism (15), dampened susceptibility to stress (21, 22), and improved learning discrimination in rodents (23).

These observations suggest that prebiotic manipulation of the microbiota–gut–brain axis could directly impact both homeostatic and non-homeostatic control of food intake. However, the behavioral and molecular consequences of prebiotic supplementation onto the reinforcing and motivational components of food seeking have hitherto been largely unexplored.

In the current study, we investigated how administration of FOS could oppose-in a preventive-like approach or reverse-in a curative approach the behavioral and molecular adaptations induced by high-fat high-sucrose exposure and their consequences on food preference and motivation for food seeking.

## Materials and Methods

### Animals and Diets

Ten-weeks-old male mice C57Bl/6J (25–30 g, Janvier, Le Genest Saint Isle, France) were housed in stainless steel cages in a room maintained at 22 ± 1°C with light from 7:00 a.m. to 7:00 p.m. Food (Safe, Augy, France) and water were given *ad libitum* unless otherwise stated. C57Bl/6J were split in six groups (*n* = 12/group). The first four groups were exposed, respectively, during 2-months to a control diet (Ctrl, 3,438 kcal/kg, protein 19%, lipid 5%, carbohydrates 55%, reference #U8959 version 63 Safe, Augy, France), a control diet enriched in fructo-oligosaccharide (Ctrl-FOS, 3,438 kcal/kg, protein 17%, lipid 8%, carbohydrates 49%, oligofructose 10%), a high-fat high-sugar diet (HFHS, 4,362 kcal/kg, proteins 20%, lipid 23%, carbohydrate 37%, reference #U8954 version 14 Safe, Augy, France), and an HFHS diet enriched in fructo-oligosaccharide (HFHS-FOS, 4,362 kcal/kg, proteins 18%, lipid 20%, carbohydrate 34%, oligofructose 10%). The last two groups were subjected to a 2-months HFHS diet and split in two groups (*n* = 12) that received during the following 2-months a “control” diet (HFHS/Ctrl) and a control diet enriched in fructo-oligosaccharide (HFHS/Ctrl-FOS). Groups supplemented with FOS will also be referred in the text by preventive (Ctrl-FOS, HFHS-FOS) or corrective (HFHS/Ctrl-FOS) effects of FOS. All animal experiments were performed with approval of the Animal Care Committee of the University Paris Diderot-Paris 7 and according to European directives.

### Body Composition Analysis

Mice were monitored for body weight and composition at the beginning and the end of the experiment. Body mass composition (lean tissue mass, fat mass, free water, and total water content) was analyzed using an Echo Medical systems’ EchoMRI (Whole Body Composition Analyzers, EchoMRI, Houston, TX, USA), according to manufacturer’s instructions.

### Measurement of Food Intake and Food Preference

Analyses were performed in an automated online measurement system using high sensitivity feeding and drinking sensors and an infrared beam-based activity monitoring system (Phenomaster, TSE Systems GmbH, Bad Homburg, Germany).

Mice were evaluated for food preference when exposed to HFHS and control chow diet (CTRL). Food preference was measured during six short sessions of 2 h (days 1–6) using animals as their own controls.

### Operant Conditioning System

Operant responding performance was performed as previously described (24). Computer-controlled operant conditioning was conducted in 12 identical conditioning chambers equipped with a swiveling infusion device (Phenomaster, TSE Systems GmbH, Bad Homburg, Germany). Each chamber contains an operant wall with a food cup, two levers located 3 cm lateral to the food cup, with the left lever designated the active lever (for food pellet delivery). Mice are maintained at 90% of initial body weight to facilitate initial learning and performance of a fixed ratio (FR1) operant learning task. The reinforcer was a single 20-mg peanut butter flavored sucrose tablet (TestDiet, Richmond, VA, USA).

Operant training was carried out over six consecutive days with two overnight fix ratio of 1 (FR) and then four consecutive days with one 2-h trial of FR1 per day. At the conclusion of the 6-days operant training period, animals were given four trials to lever press for sucrose under a progressive ratio of 3 (PR), lever press requirement for each subsequent reinforcer increased by 3 with an initial requirement of 3 lever press (*r* = 3*N* + 3; *N* = reinforcer number).

The PR schedule requires the mouse to perform an increasing number of lever presses for each consecutive reward, the number of rewards received (also called breakpoint) was used to assess motivation or effort to work for a food reward.

At the end of the experiment, animals were sacrificed, brain, liver, cecum tissues, and plasma collected.

### Gut Microbiota Analysis

At the end of the experiment, the total cecum content was collected and weighed before storage at −80°C. Metagenomic DNA was extracted from the cecal content using the QIAamp DNA stool mini kit (Qiagen, Hilden, Germany) according to the manufacturer’s instructions. Quantitative PCR (qPCR) for total bacteria, *Bifidobacterium* spp., *Lactobacillus* spp., *Akkermansia muciniphila, Roseburia* spp., and *Bacteroides-Prevotella* spp. were performed by using Mesa Fast qPCR™ (Eurogentec, Seraing, Belgium). Real-time PCRs were performed with the StepOnePlus™ real-time PCR system and software (Applied Biosystems, Den Ijssel, The Netherlands). The primers sequences were described previously (15, 25). Cycle threshold of each sample was then compared with a standard curve (performed in triplicate) made by diluting genomic DNA obtained from BCCM/LMG (Ghent, Belgium) or DSMZ (Braunshweig, Germany). Prior to isolating the DNA, the cell counts were determined by BCCM/LMG or DSMZ, respectively; fivefold serial dilution of *Bifidobacterium animalis* BCCM/LMG 18900 for *Bifidobacterium* spp., *Bacteroides fragilis* BCCM/LMG 10263 for *Bacteroides-Prevotella* spp., *Lactobacillus acidophilus* DSM 20079 for *Lactobacillus* spp., *A. muciniphila MucT* (ATTC BAA-835, DSMZ22959) for *A. muciniphila, Roseburia intestinalis* (DSMZ 14610) for *Roseburia* spp., and *Lactobacillus acidophilus* DSM 20079 for total bacteria.

### Isolation of Total RNA and Quantitative RT-PCR

From all groups, total hypothalamic RNA was extracted and analyzed by qRT-PCR for agouti-related protein (AgRP), neuropeptide Y (NPY), pro-opiomelanocortin (POMC), and cocaine and amphetamine-regulated transcript (CART), and total nucleus accumbens (NAcc) RNA was extracted and analyzed by qRT-PCR for dopamine transporter (DAT), dopamine receptor D1 (DR1), dopamine receptor D2 (DR2), dopamine beta hydroxylase (DBH), and tyrosine hydroxylase (TH).

Total RNA was isolated as described previously (9). We retro transcribed 1 µg RNA using Superscript II (Invitrogen). Real-time quantitative (qRT-PCR) analyses were performed with 25 ng cDNA and 250 nM sense and antisense primers (Eurogentec) in a final reaction volume of 25 µl by using qPCR Core Kit (Eurogentec) and the MyiQ real-time PCR detection system (Bio-Rad). Specific primers were designed using Primer Express software (version 1.0, Applied Biosystems) and primers sequences and housekeeping gene (HKG) are listed below. Relative quantification of hypothalamic and NAcc RNA for each gene was calculated after normalization to HKG by using the comparative Ct method.

**Table:** 

Primers	Sequences
POMC	Forward: 5′-AGTGCCAGGACCTCACCA-3′ Reverse: 5′-CAGCGAGAGGTCGAGTTTG-3′
NPY	Forward: 5′-CCGCTCTGCGACACTACAT-3′ Reverse: 5′-TGTCTCAGGGCTGGATCTCT-3′
AgRP	Forward: 5′-CGGAGGTGCTAGATCCACAGA-3′ Reverse: 5′-AGGACTCGTGCAGCCTTACAC-3′
CART	Forward: 5′-CGAGAAGAAGTACGGCCAAG-3′ Reverse: 5′-CTGGCCCCTTTCCTCACT-3′
DAT	Forward: 5′-GCCCTACCTGCTCTTCATGC-3′ Reverse: 5′-GGATGACAGTGAAGCCCACA-3′
DR1	Forward: 5′-TCTGGTTTACCTGATCCCTCA-3′ Reverse: 5′-GCCTCCTCCCTCTTCAGGT-3′
DR2	forward: 5′-TGAACAGGCGGAGAATGG-3′ Reverse: 5′-CTGGTGCTTGACAGCATCTC-3′
DBH	Forward: 5′-ATCTCCATGCATTGCAACAA-3′ Reverse: 5′-AGGCTGCAGATTCCACTCAC-3′
TH	Forward: 5′-GGTATACGCCACGCTGAAGG-3′ Reverse: 5′-TAGCCACAGTACCGTTCCAGA-3′
RPL19 HKG	Forward: 5′-GGGCAGGCATATGGGCATA-3′ Reverse: 5′-GGCGGTCAATCTTCTTGGATT-3′
H1A HKG	Forward: 5′-AGAAGAACAACAGCCGCATC-3′ Reverse: 5′-TGCACCAGTGTGCCTTTATT-3′
HPRT HKG	Forward: 5′-GTTGGATACAGGCCAGACTTTGTTG-3′ Reverse: 5′-GATTCAACTTGCGCTCATCTTAGGC-3′
CYCLOA HKG	Forward: 5′-ACGCCACTGTCGCTTTTC-3′ Reverse: 5′-GCAAACAGCTCGAAGGAGAC-3′

### Statistical Analysis

Displayed values are mean ± SEM. Variance equality was analyzed by a paired *t*-test (GraphPad Prism 6^®^). Unless otherwise stated, comparisons between groups were carried out using analysis of variance (ANOVA, GraphPad Prism 6^®^). A *P*-value of less than 0.05 was considered statistically significant.

## Results

### Preventive vs Corrective Prebiotic Supplementation Differentially Impact Body Weight and Gut Microbiota Composition

**Figure 1:**
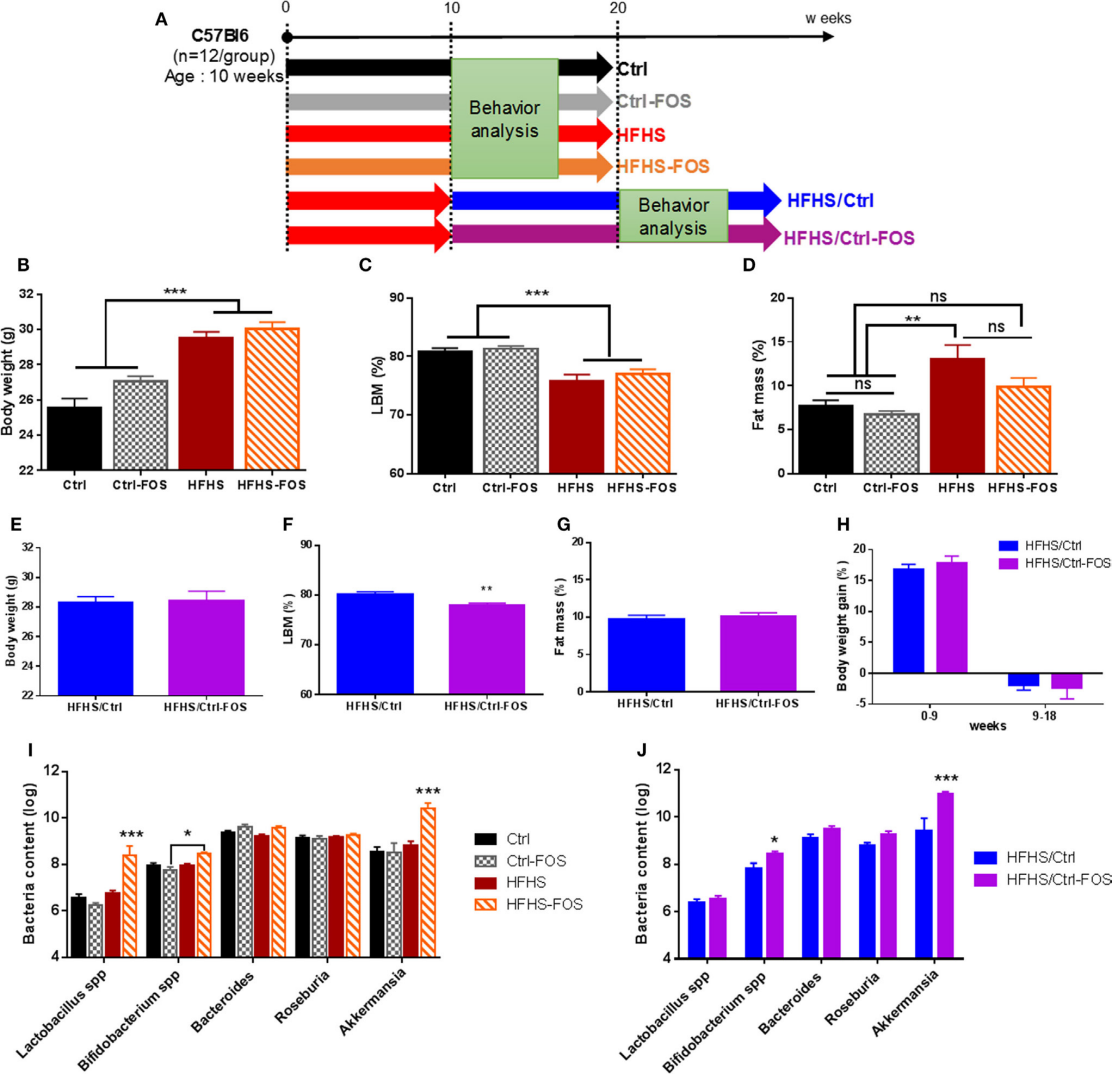
**(A)**. Experimental design of diet manipulation in which mice were fed, respectively, regular chow (Ctrl, black), high-fat high-sucrose diet (HFHS, red), or diet enriched in fructo-oligosaccharides (FOS) (Ctrl-FOS, gray and HFHS-FOS, orange) in a protective approach. Two additional groups were fed an HFHS and switched onto either regular chow diet (HFHS/Ctrl, blue) or regular chow enriched in FOS (HFHS/Ctrl-FOS, purple). **(B–H)** Body weight and body composition evolution during diet manipulation in the six different groups. **(B,E)** Body weight evolution, **(C,D,F,G)** body composition in **(C,F)** lean body mass (LBM) and **(D,G)** fat body mass in Ctrl (black), Ctrl-FOS (gray), HFHS (red), HFHS-FOS (orange), HFHS/Ctrl (blue), and HFHS/Ctrl-FOS (purple). **(I,J)** Targeted metagenomics analysis of cecum bacterial content in animal fed Ctrl (black), Ctrl-FOS (gray), HFHS (red), and HFHS-FOS (orange) and **(J)** after switch on Ctrl or Ctrl-FOS diet in HFHS/Ctrl (blue) and HFHS/Ctrl-FOS (purple). Data are expressed as mean ± SEM of 12 mice in each group. Significant differences from a one-way ANOVA **(B–D)**, paired *t*-test **(F)** and a two-way ANOVA **(I,J)**, *Bonferroni Post hoc* test are shown (*P < 0.005, **P < 0.001, ***P < 0.0005).

Four groups were subjected to a 2-months long preventing-like approach of prebiotic supplementation in which two groups received regular chow diet with or without soluble fibers FOS (Ctrl or Ctrl-FOS groups) while two last groups received a similar treatment but were raised on palatable high-fat high-sucrose diet with or without FOS (HFHS or HFHS-FOS groups) (Figure 1A). An additional two animal cohorts were dedicated to explore the corrective potency of prebiotic supplementation onto metabolic and behavioral changes induced by a 2 months exposure to HFHS diet. In this setting, 12 weeks-old C57Bl/6J male mice were first raised on HFHS diet for 2-months then shifted onto Ctrl or Ctrl-FOS diet and will be referred as to HFHS/Ctrl or HFHS/Ctrl-FOS, respectively (Figure 1A). Based on the extensive literature in the field, we chose a 10% FOS enrichment since it was described to promote metabolic benefits (26–28) together with improvement in learning discrimination and improved cognitive performances (23, 29).

While average body weight did not differ among groups before the treatment, a significant increase in body weight was reached after HFHS but not HFHS-FOS exposure (Figures 1B,E,H). Body weight gain was mostly attributable to adipose tissue as revealed by body composition analysis (Figures 1C,D,F,G; Figures S1A–D in Supplementary Material). In HFHS/Ctrl and HFHS/Ctrl-FOS groups, the switch of HFHS to control diet stopped diet-induced body weight gain (Figure 1H) in similar way between FOS and non-FOS-treated group (Figure 1H; Figure S1E in Supplementary Material).

Prebiotic supplementation is known to change the composition and activity of specific gastrointestinal microbiota (30, 31). Therefore, we decided to investigate if specific bacteria were modified following our diets. qPCR analysis of the cecum bacterial content revealed that the prevalence of *Bifidobacterium* spp., *A. muciniphila*, and to a lesser extent *Lactobacillus* spp. was significantly increased but only in condition in which both prebiotics and HFHS were combined either simultaneously or when FOS supplementation followed HFHS exposure. It is worth to mention that other bacterial families, such as *Roseburia* spp. and *Bacteroides* spp., were not affected by dietary regimens (Figures 1I,J). However, when mice were exposed to Ctrl or Ctrl-FOS diets, we could not identify significant changes in the microbiota ecosystem (Figures 1I,J).

### Timing in Prebiotic Supplementation Is Instrumental in the Beneficial Impact on Hedonic and Motivational Component Feeding

**Figure 2:**
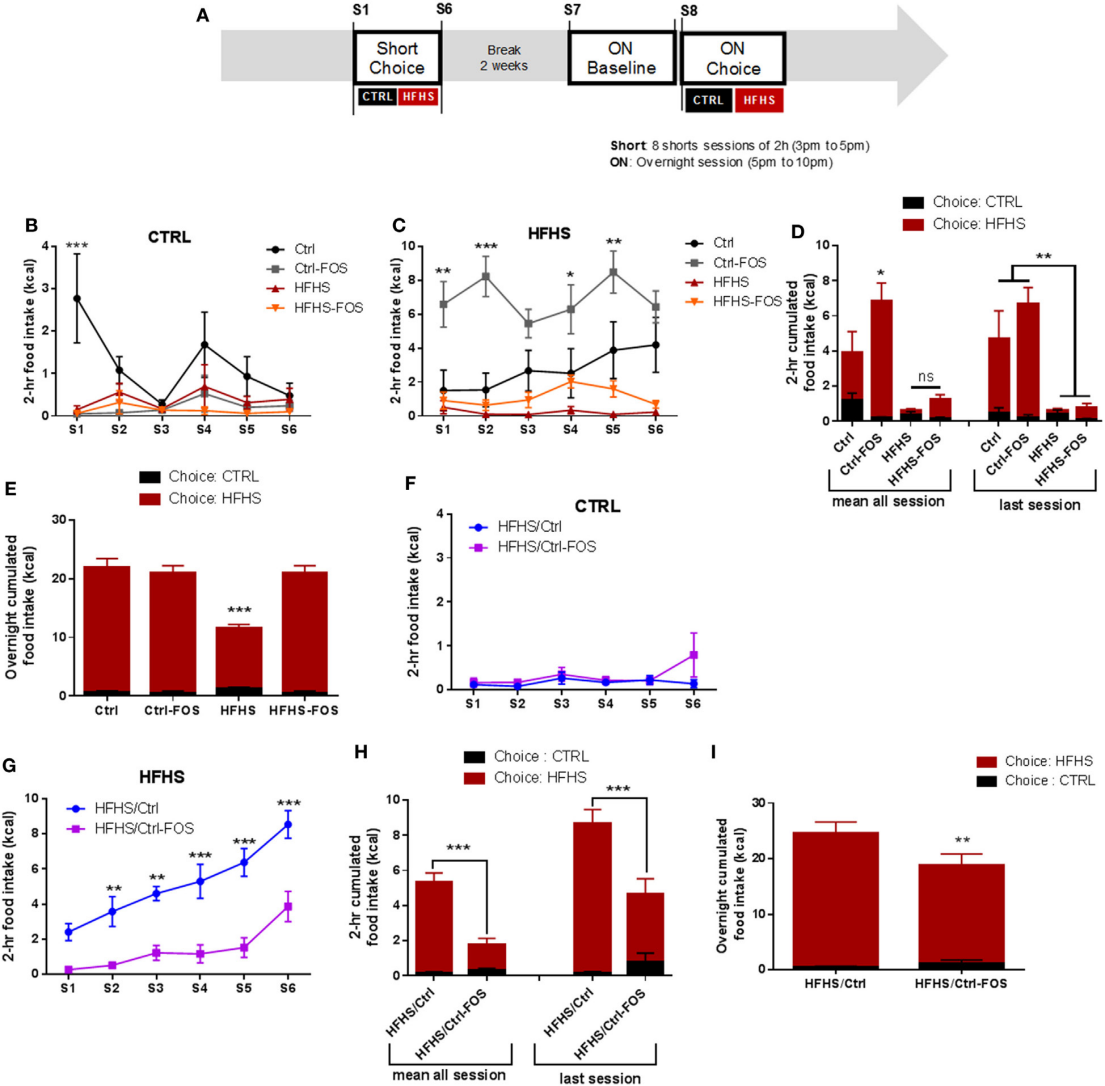
**(A)** Experimental design for food tropism analysis using short or overnight access to a two-food choice protocol using regular chow diet or palatable HFHS diet for six mice per group. Regular chow diet [CTRL **(B,D,F,H)**] or HFHS intake **(C,D,G,H)** during seven sessions consisting of a 2-h two-food choice access, **(D,H)** representative tropism for CTRL *vs* HFHS as average cumulated intake through all session or during the last session, **(E,I)** representative tropism for CTRL vs HFHS as average cumulated intake overnight in Ctrl (black), Ctrl-fructo-oligosaccharides (FOS) (gray), HFHS (red), HFHS-FOS (orange), HFHS/Ctrl (blue), and HFHS/Ctrl-FOS (purple). Data are expressed as mean ± SEM of six mice in each group. Significant differences from a two-way ANOVA, *Bonferroni Post hoc* test (*P < 0.005, **P < 0.001, ***P < 0.0005) **(B–I)**.

Previous data suggest that modifications in the microbial diversity may influence food choice and tropism in the host and participate to satiety responses by regulating the gut–brain axis (16, 30, 32). After nutritional manipulation aimed at either preventing or correcting any metabolic and behavioral changes induced by HFHS exposure, the six groups were subjected to a two-food choice paradigm consisting in a seven daily consecutive 2 h-limited access to both Ctrl and HFHS diet followed by an overnight exposure to food choice (Figure 2A). Short-term access to two-food choice aimed at deciphering the preference to select and consume palatable over chow pellets in a non-fasted condition. While Ctrl-fed animals displayed a tropism for HFHS over control chow diet (CTRL), Ctrl-FOS maximized their consumption of HFHS starting from the first session (Figures 2B–D). During the overnight exposure to food choice, a similar pattern was observed with increased HFHS consumption in Ctrl and Ctrl-FOS animals with an overall 90% preference for the palatable diet (Figure 2E).

Interestingly both cohorts exposed to HFHS diet (HFHS and HFHS-FOS) displayed minimal consumption of palatable diet when given the choice on a short period of time (Figures 2B–D), in agreement with the reduction in palatable food preference observed in animal fed with high-fat diet (33). This result contrasted, however, with a large preference for HFHS over CTRL in the overnight access in both HFHS and HFHS-FOS groups (Figure 2E). In addition, FOS supplementation in HFHS-FOS led to increased tropism for palatable diet compared to HFHS group (Figure 2E). These results indicate that mice exposed to diets supplemented with FOS (Ctrl or HFHS) show increased preference for palatable food (Figures 2B–E).

In sharp contrast with the lack of preventive action, when prebiotics were added in the diet after a 2 months HFHS exposure (HFHS/Ctrl-FOS), we observed a strong corrective action of FOS on both 2-h time-restricted and overnight palatable diet intake (Figures 2F–I).

**Figure 3:**
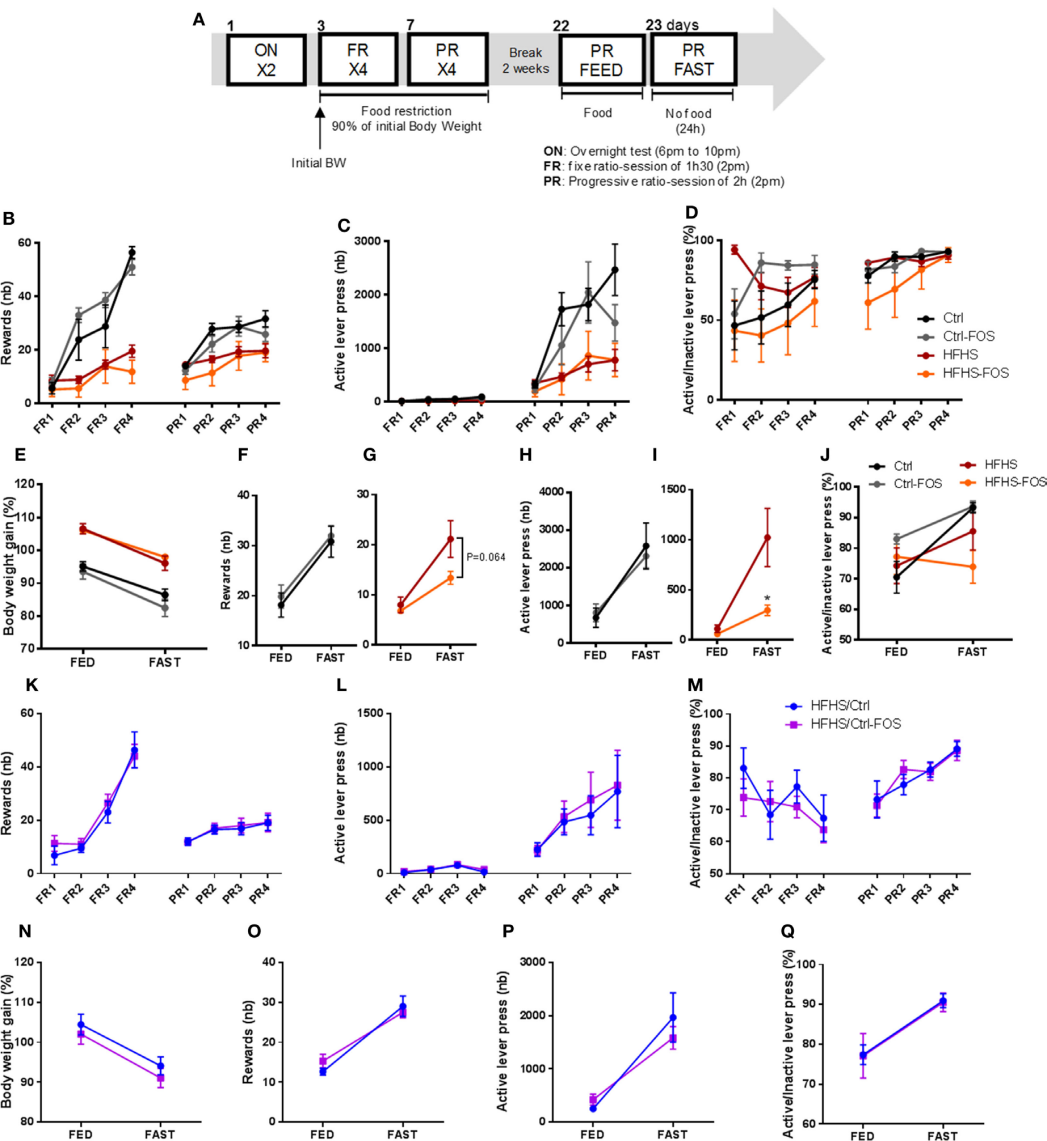
**(A)** Experimental design for the operant responding performance assessment for six mice per group. **(B,K)** Reward number, **(C,L)** active lever press, **(D,M)** ratio between the active and inactive lever presses in **(B–D)** Ctrl (black), Ctrl-fructo-oligosaccharides (FOS) (gray), HFHS (red), **(K–M)** HFHS-FOS (orange), HFHS/Ctrl (blue), and HFHS/Ctrl-FOS (purple) during a 90% body weight reduction. **(E,N)** body weight change, reward number **(F,G,O)**, active lever press **(H,I,P)** or active vs inactive lever press ratio **(J,Q)** in response to an overnight fast in Ctrl (black), Ctrl-FOS (gray), HFHS (red), HFHS-FOS (orange), HFHS/Ctrl (blue), and HFHS/Ctrl-FOS (purple). Data are expressed as mean ± SEM of six mice per group. Significant differences from a two-way ANOVA, *Bonferroni Post hoc* test are shown (*P < 0.005) **(I)**.

Food preference and seeking strongly rely on dopamine whose release within the mesocorticolimbic (MCL) system participate in driving the motivational and reinforcing values of food reward (6, 34–36). Hence, we next sought to behaviorally dissect the consequences of FOS supplementation on the motivational drive to obtain food rewards. After nutritional manipulation, the different cohorts underwent through an operant conditioning task to obtain food rewards. Animals were first subjected to fixed ratio (FR) reinforcement schedule in which a single lever press triggers the delivery of a palatable food pellet. Once mice have reached their discriminatory ability between active and inactive lever, they are shifted to a progressive ratio (PR) in which the number of lever presses required to obtain a reward increases progressively (Figure 3A). If a subject abnormally inflates the reinforcing aspect of a reward it will be more likely to exert effort to obtain it. Alternatively, if the perceived value of the reward is abnormally diminished, the willingness to engage in effortful behavior to obtain it will be reduced. In all conditions, operant responding for sucrose pellets reward was evaluated on mice gradually food restricted to 90% of body weight or following acute overnight in fed or fasting condition (Figure 3A).

Here again, the behavioral output of prebiotic supplementation was different according to the timing (preventive or corrective) of FOS addition in the diet. Ctrl and Ctrl-FOS groups had a similar profile with enhanced operant responding for food reward compared to both HFHS and HFHS-FOS group (Figure 3B). These results are consistent with the previous studies showing attenuated operant performance in high-fat fed animals (37). Surprisingly, FOS addition did not alter the number of collected rewards (Figure 3B), active lever press (Figure 3C), and discriminatory capacity between active and inactive lever (Figures 3D,J) in animal exposed to Ctrl diet (Ctrl and Ctrl-FOS) under both chronic and acute fasting-induced body weight loss (Figures 3E,F,H). However, FOS supplementation on HFHS diet decreased the motivational drive to collect food reward under conditions of drastic energy deprivation (Figures 3G,I).

A similar experimental design was carried out to evaluate operant conditioning in animals previously fed with an HFHS diet and shifted under Ctrl or Ctrl-FOS diets (HFHS/Crtl, HFHS/Ctrl-FOS).

While a corrective property of FOS supplementation was evident in palatable diet intake and preference (Figures 2F–I), HFHS/Crtl and HFHS/Ctrl-FOS exhibited identical performance in every aspect of operant response for food reward in both tested conditions (chronic or acute food deprivation) (Figures 3K–Q).

These results highlight that prebiotics might exert a distinct and specific action onto the hedonic “liking,” and motivational “wanting” drive to consume palatable food as well as feeding response to energy deprivation. Furthermore, our data support the notion that time-dependent exposure to prebiotics may be instrumental in their action onto reward-seeking behavior.

### Timing in Prebiotic Supplementation Is Instrumental in Molecular Adaptation in Mesolimbic and Hypothalamic Structures

The action of feeding results from the ability of the brain to properly integrate circulating signals of hunger and satiety together with food-related cues coding for palatable and rewarding values (38). The hypothalamus–brainstem axis, by primarily encoding metabolic needs, is regarded as the key neural network in the homeostatic control of body weight whereas the dopaminergic system is mainly involved in encoding the rewarding and reinforcing values of food seeking. Hence, hypothalamic–brainstem circuit is typically referred as to homeostatic while mesolimbic circuit are referred as to non-homeostatic regulation of feeding (38, 39). Interestingly, obesity and high-fat feeding have been shown to provoke various adaptive changes in both MCL and hypothalamic structures which could account for the toxic effect of energy-dense food (8, 10, 40–43).

**Figure 4:**
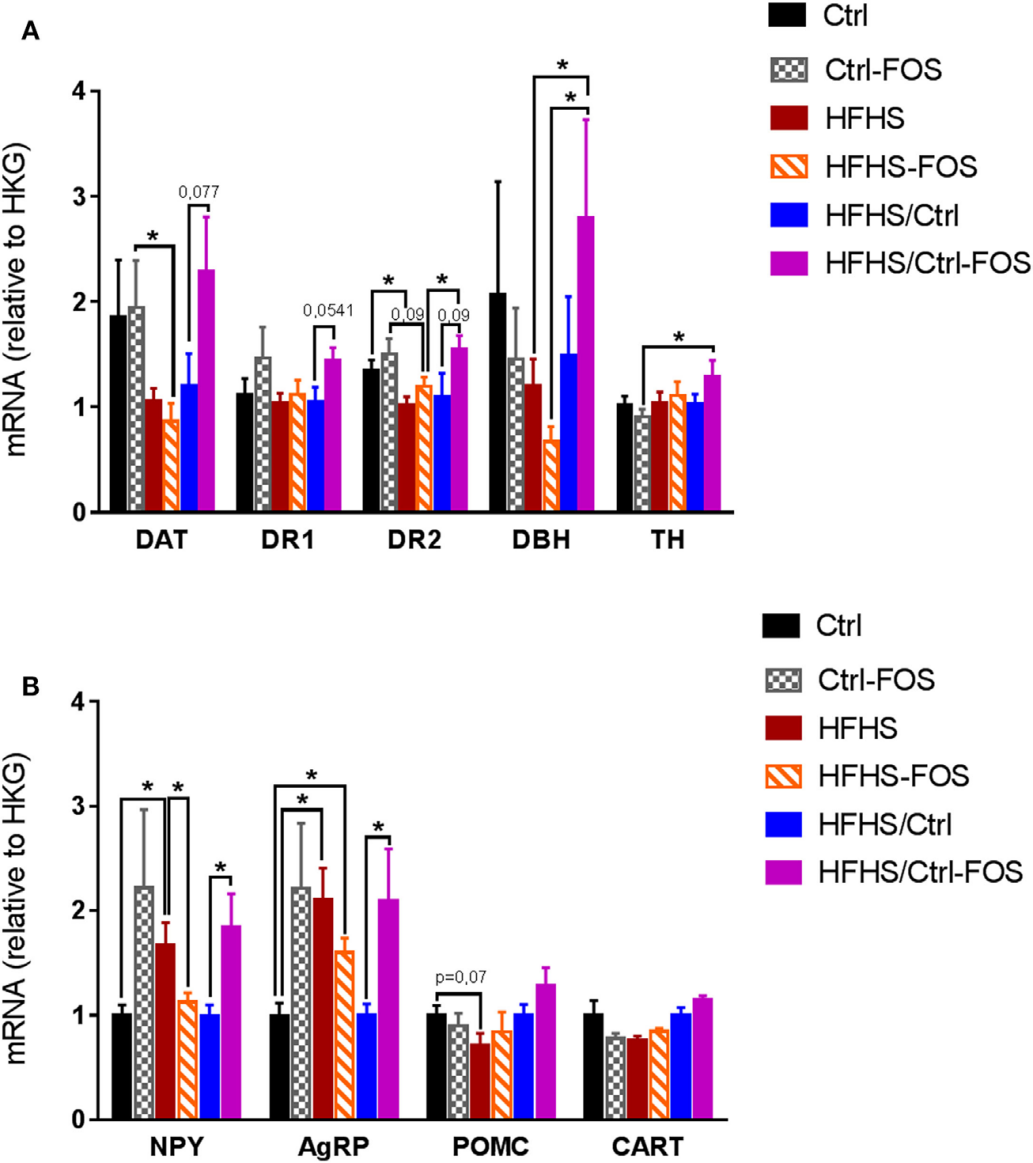
Real-time PCR analysis of mRNA content **(A)** in the nucleus accumbens, for gene encoding for dopamine transporter (DAT), dopamine receptor 1 (DR1), dopamine receptor 2 (DR2), dopamine beta hydroxylase (DBH), and tyrosine hydroxylase (TH) and **(B)** in the hypothalamus for neuropeptide Y (NPY), Agouti-related protein (AgRP), Pro-opiomelanocortin (POMC), and cocaine and amphetamine-regulated transcript (CART) in Ctrl (black), Ctrl-fructo-oligosaccharides (FOS) (gray), HFHS (red), HFHS-FOS (orange), HFHS/Ctrl (blue), and HFHS/Ctrl-FOS (purple). Expression level corresponds to a ratio relative to housekeeping gene (HKG). Data are expressed as mean ± SEM of eight mice in each group. Significant differences from a two-way ANOVA, *Bonferroni Post hoc* test (*P < 0.005) **(A,B)**.

We therefore explored how time-dependent prebiotic manipulation modulated the molecular adaptations of dopaminoceptive and hypothalamic structures in response to high-fat feeding. mRNA were extracted from the NAcc and hypothalamus and analyzed for expression of genes involved in dopamine synthesis and signaling, i.e., DAT, DBH, DR1, DR2, and TH in the NAcc, while genes encoding neuropeptides involved in melanocortin signaling and body weight regulation i.e., NPY, agouti-related protein (AgRP), POMC, and cocaine and amphetamine-regulated transcript (CART) in the hypothalamus (Figures 4A,B).

In a preventive approach, the effect of FOS addition to Ctrl or HFHS diets was evident in hypothalamic expression of energy-related neuropeptides but failed to alter expression of dopamine-associated genes in the NAcc. Both Ctrl and Ctrl-FOS displayed similar levels of mRNA encoding DAT and DR2 that were significantly higher than those observed in either HFHS or HFHS-FOS (Figure 4A). In the hypothalamus, however, while exposure to energy-dense food led to increased mRNA contents for NPY and AgRP in HFHS group, prebiotic supplementation induced a significant decrease in NPY expression (Figure 4B). These results are in agreement with published observations showing that high-fat diet and/or obesity result in decreased expression of DR2 and DAT (7, 8) and, on the one hand, increased expression of hypothalamic orexigenic peptides (44, 45). Our results show that, in a preventive-like approach, FOS supplementation partially restores hypothalamic expression of orexigenic peptides but fails to correct the modifications induced by energy-dense food in the NAcc (Figures 4A,B).

Surprisingly, FOS enrichment had an opposite consequence onto hypothalamic peptides on mice exposed to Ctrl or HFHS diets. Ctrl-FOS diet led to increases in both orexigenic peptides NPY and AgRP compared to Ctrl group while the same nutritional manipulation operated onto HFHS diet led to a decrease of these peptides (Figure 4B). In both conditions, the modulation of NPY and AgRP were not counterbalanced by a change in the expression of the anorectic transcripts for POMC or CART (Figure 4B).

This result provides a molecular underpinning supporting the relative hyperphagia observed in Ctrl-FOS compared to Ctrl animals in food choice condition (Figures 2C,D). In the same food choice paradigm, FOS enrichment in HFHS diet led to increased consumption of palatable diet in an overnight session (Figure 2E) which point toward a decorrelation between hypothalamic decrease in NPY, AgRP and food reward seeking.

In a corrective-like approach, however, prebiotic supplementation to Ctrl diet in animal previously exposed to HFHS diet for 2 months fully restored Nacc level of DAT, DR2, and DBH (Figure 4A). However, while the shift onto Ctrl diet was *per se* sufficient to restore normal hypothalamic levels for NPY and AgRP, this benefic action was counterbalanced by FOS addition which was associated to sustained level of both orexigenic peptides (Figure 4B).

Altogether our results show that the molecular adaptations induced by high-fat feeding in brain structures that govern food intake in response to either metabolic demand or reward can be restored or opposed by prebiotic supplementation. Importantly, the timing in FOS supplementation together with the nature of the diet in which FOS is introduced with have critical impact on the direction by which prebiotic will operate the adaptive changes in MCL or hypothalamic structures and ultimately predict the ability of prebiotic to change food tropism and reward-seeking behavior.

## Discussion

While metabolic needs are primarily encoded in the hypothalamus, the reinforcing value of food encompasses a multisensory component including flavors and texture which ultimately modulates the release of DA in the MCL system. In modern society, calorie-dense foods are widely available and were associated with the progression of obesity together with the development of compulsive eating in which reward-driven eating behaviors are bypassing homeostatic regulation of nutrients intake (4, 46, 47). The microbiota–gut–brain axis has emerged as a pivotal player in appetite control as well as reward-driven behavior (48).

In the present study, we described the impact of prebiotic supplementation onto various components of food reward-seeking behavior, gut microbiota ecosystem and molecular adaptation in both hypothalamic and mesolimbic structures. We used food choice paradigm associated with operant conditioning to lever press for food reward in order to dissect out how FOS supplementation could prevent or correct the consequence of chronic exposure to palatable, energy-rich diet onto the hedonic and motivational component of food seeking behavior. We manipulated the timing of FOS introduction in the diet using, first, a preventive-like approach in which animals were exposed to CTRL diet or HFHS diet with or without FOS supplementation and second, a corrective-like approach in which animal were first raised on HFHS diet and then switched onto CTRL diet with or without FOS. In both cases, the consequences onto gut microbiota, hedonic and motivational aspect of food reward together with brain expression of genes involved homeostatic and non-homeostatic control of feeding.

We found that prebiotics act in synergy with the diet supplied to operate change in microbiota composition, tropism for palatable food, and hypothalamic and MCL response. Using targeted metagenomics approach, we could only identify selected changes in the gut–microbiota ecosystem, especially modifying the contents of *Bifidobacterium* spp., *A. muciniphila*- and *Lactobacillus* spp., but only in animals that were either raised or had been exposed chronically to HFHS diet (Figures 1I,J). In the same line we found that, while FOS addition to CTRL diet increased both the drive for palatable diet (Figures 2C–E) and hypothalamic expression of orexigenic neuropeptides NPY and AgRP (Figure 4B), prebiotic addition decreased the motivation to collect food rewards after a fast and decreased hypothalamic NPY content in HFHS fed animals (Figures 3G,I and 4B).

Surprisingly enough, in our hands FOS introduction to the diet only modestly affected HFHS-induced fat mass gain (Figure 1D; Figure S1B in Supplementary Material) but had no significantly impact of body weight gain or body weight loss after the transition from HFS to CTRL diet (Figures 1E–H; Figures S1C–E in Supplementary Material). However, despite the lack of effect on body weight, we could clearly demonstrate that the timing of prebiotic supplementation had a pivotal role in both molecular and behavioral responses in food reward seeking and consumption. After a 2-months HFHS exposure, we tested the capacity of prebiotic to revert molecular and behavioral dysfunctions induced by caloric overload. The shift onto CTRL diet similarly surfeited body weight gain regardless of FOS addition, however, in contrast to the chronic preventive approach, prebiotic supplementation resulted in decreased palatable food tropism and consumption (Figures 2F–I) without affecting operant performance (Figures 3K–Q) and was associated with concomitant increase in hypothalamic orexigenic markers and NAcc expression of gene involved in DA signaling. This latter result suggests that, unlike the preventive addition of FOS, prebiotic treatment after chronic HFHS exposure helped restoring the imbalance in MCL DA signaling and reward-driven tropism and overconsumption of palatable diet. Importantly, these changes primarily affected hedonic rather than motivational aspects of food reward and had a positive impact on food choice despite increased expression of hypothalamic orexigenic neuropeptides.

It is important to note that, while 10% FOS supplementation correlated with positive change in the gut microbiota ecosystem as expected from the literature, we did not observe a clear benefit on body weight. FOS introduction did mitigate fat mass gain in animal raised onto HFHS diet (Figure 1D) but on overall did not significantly modify body weight. Of note, however, while a decrease in body weight could be expected from prebiotic treatment it is important to highlight that while FOS supplementation has been shown to increase post-meal satiety and hunger, change in body weight were not always consistently observed. Indeed it when compared to other dietary fibers FOS supplementation was associated with body weight gain in lean rodents (28) and obese mice (49) while other report clearly show a preventive action of FOS on high-fat-mediated body weight gain (50). A genetic model of metabolic syndrome FOS supplementation was shown to drastically alleviate excessive feeding but had no impact on body weight (29). This study comes in addition with several report that clearly established the benefits of FOS onto glucose control and insulin sensitivity (51) and in that regards it is tempting to speculate that enhanced insulin sensitivity (29), while promoting a more healthy adipose development, could mitigate the overall body weight loss.

Despite overall similar body weight in within cohort, we found very different outcomes at the behavioral and molecular level when FOS supplementation was added during or after HFHS diet exposure. We first described a paradoxical action of FOS when assessed onto CTRL diet that increased the tropism for palatable diet when assessed on a two-food choice paradigm (Figures 2B–D) and while this result is in good agreement with the increase in hypothalamic orexigenic peptides (Figure 4B) it could also potentially be the consequence of anxiolytic-like properties of prebiotic (21) which might alleviate food neophobia classically observed in C57BL6 mice (29, 52) and result in faster maximization of palatable diet intake when given the choice. Importantly, however, it should be noted that our behavioral assay was designed to address how animals spontaneously prefer, or are willing to work for food reward and although alteration on reward feeding might lead to overconsumption (46), our protocol does not provide a measure on the long-term consequence onto body weight.

Chronic palatable diet exposure has been shown to promote changes in the reward system at both molecular and behavioral levels (8, 10, 46). One possible explanation for this timing effect of prebiotic action that we observed might be encapsulated in the fact that pre exposure to energy-dense food might initiate both peripheral and central adaptive changes among which some could be selectively corrected by FOS addition. Indeed, once rodent have been exposed to reinforcing stimulus such as palatable diet or drug of abuse they are typically more prone to develop addictive-like behavior (8, 46). These adaptive changes can involve one or many components of the DA system (7, 8, 46, 53) in association with alteration of the gut microbial ecosystem (54). For instance, energy-dense food exposure leads to diet-induced central inflammation (55), neuropeptide signaling alteration (29, 44), and decrease in dopamine receptor abundance (8, 10, 46), which would presumably participate in the development of addictive/compulsive eating behavior. Aside of a direct action onto the brain, energy-dense food also target the gut to control reward acquisition. Gut detection of dietary lipids have been shown to directly control DA release and action by route of the vagal nerves (56). These regulatory processes are probably part of larger integrative aspects by which the combination of diet and microbiota can influence host appetite through change in gut-derived metabolite, intestinal barrier, immune system (48).

Hence, the combination of HFHS exposure followed by prebiotic addition might overall change the microbia–gut–brain axis resulting in the fine-tuning or resetting of DA signaling and reward-driven behavior. Indeed, when FOS was added after HFHS exposure, we could observe a restoration of mesolimbic markers of DA signaling (Figures 4A,B) and, despite the increase in orexigenic NPY and AgRP observed the HFHS/CTRL-FOS group displayed strong reduction in food reward tropism (Figures 2F–H). This points at a rather dominant function of the reward system in the control of feeding in animals pre-exposed to palatable diet. Interestingly, the study from de Cossio and colleagues also described a beneficial action of prebiotic onto hyperphagia in obese animals that was independent of any changes in NPY, POMC was blunted by prebiotic addition, hypothalamic neuropeptide related (29).

Our results suggest that manipulating of the gut–brain axis can, in specific condition, exert a satietogenic effect primarily by modulating hedonic and motivational drive for food reward. This is in good agreement with the emerging concept that micriobiota–gut–brain axis is a potential avenue to modulate reward and in general addictive behavior (57, 58). Notwithstanding, a great limitation of our study lies in the use of targeted metagenomics approach that only accounted for specific bacterial strain changes. It is clear that prebiotic treatment will have consequences on gut flora that extend far beyond the changes that we described here (Figures 1I,J) and it is formally possible that one or multiple changes in the gut ecosystem that were not addressed here might reveal potential molecular underpinning by which bacterial–host interaction alters food reward.

In conclusion, our study depicts how timely controlled prebiotic manipulation can differentially and selectively affect positive reinforcement and motivational aspects of food reward-seeking behavior and demonstrate the efficacy of the gut–microbiota–brain axis to operate molecular adaptations in neural substrates involved in both homeostatic and non-homeostatic control of body weight. However, further studies will be warrant to precisely describe the molecular underpinning of the bacterial–host interaction in the control of food reward.

## Ethics Statement

All animal experiments were performed with approval of the Animal Care Committee of the University Paris Diderot-Paris 7 and according to European directives.

## Author Contributions

A-SD performed all the studies. JC, RD, CM, and MQ provided technical and conceptual support for behavioral and metabolic analysis and PCR analysis. AE and PC provide analysis of microbiota composition and conceptual support. FM and SL designed the study, secured the funding, and wrote the manuscript.

## Conflict of Interest Statement

FM was employed by company KOT CEPRODI. The funders had no role in study design, data collection and analysis, decision to publish, or preparation of the manuscript. All authors declare no conflict of interest and competing interests.
