# Supplementary figures and images for: Prebiotics Supplementation Impact on the Reinforcing and Motivational Aspect of Feeding
Source: Front Endocrinol (Lausanne). 2018 May 29;9:273. doi: 10.3389/fendo.2018.00273 (PMC5987188; doi:10.3389/fendo.2018.00273)

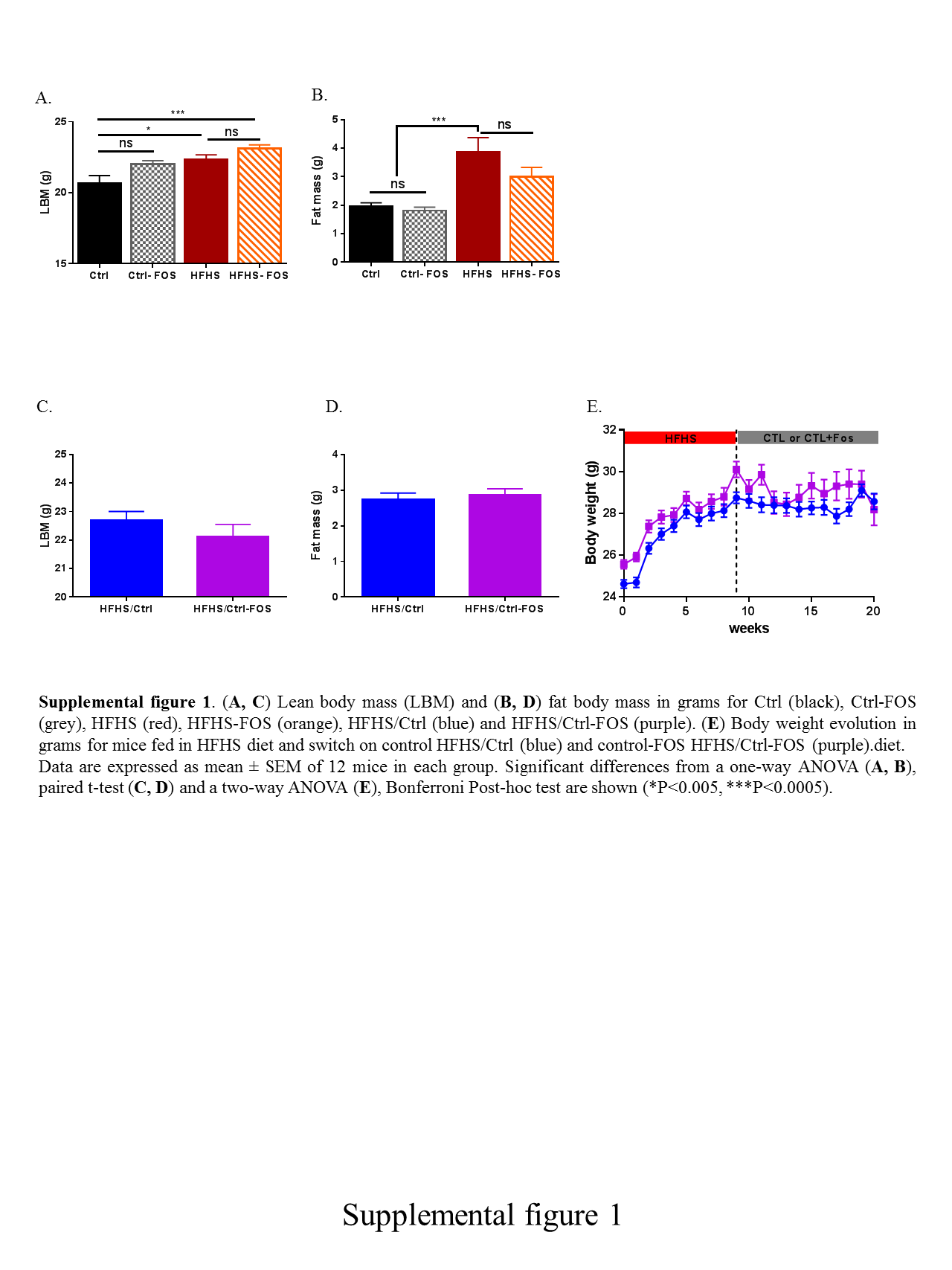

Supplement: Supplementary file 1 [file image_1.tif]
